# Lumateperone Safety and Tolerability in Schizophrenia: A Narrative Review

**DOI:** 10.7759/cureus.96165

**Published:** 2025-11-05

**Authors:** Jamal Montaser, Srihas Tumu, Venkata Yashashwini Maram Reddy, Navod Jayasuriya, Muaz Ali, Abdaal Munir, Moaz Elmontaser

**Affiliations:** 1 Psychiatry and Behavioral Sciences, California Institute of Behavioral Neurosciences & Psychology, Fairfield, USA; 2 Internal Medicine, Guntur Medical College, Guntur, IND; 3 Anesthesiology and Perioperative Medicine, Faculty of Medicine, University of Ruhuna, Galle, LKA; 4 Medicine, Rawalpindi Medical University, Rawalpindi, PAK; 5 Surgery, College of Physicians and Surgeons Pakistan, Islamabad, PAK; 6 General Surgery, Wirral University Teaching Hospital NHS Foundation Trust, Upton, GBR; 7 Human Kinetics, University of Windsor, Windsor, CAN

**Keywords:** antipsychotic side effect, atypical antipsychotic, caplyta, lumateperone, schizophrenia, second generation antipsychotics

## Abstract

Schizophrenia remains challenging to manage, as most available antipsychotic medications alleviate symptoms but are associated with significant adverse effects such as weight gain, sedation, and extrapyramidal symptoms (EPS). Lumateperone, a recently approved antipsychotic, has attracted attention due to its unique pharmacological profile. In addition to modulating dopamine receptors, it also influences serotonin and glutamate neurotransmission, potentially addressing a broader spectrum of symptoms, including cognitive and social deficits. This narrative review synthesizes recent clinical evidence on lumateperone, evaluating study design, outcomes, and consistency across trials. Current findings suggest that lumateperone reduces core symptoms of schizophrenia while demonstrating a more favorable safety profile than many established antipsychotics. In particular, it appears to carry a lower risk of metabolic and motor side effects, which may support improved long-term adherence. Overall, this review aims to contextualize the emerging body of evidence and to evaluate the potential role of lumateperone, particularly for patients with inadequate response to conventional antipsychotics.

## Introduction and background

Schizophrenia is a complex psychiatric disorder that disrupts normal patterns of thought, emotion, and behavior. It typically begins in early adulthood and is rare before age 16 [1]. Although the prevalence is less than 1% of the population, schizophrenia imposes a substantial burden due to long-term disability and reduced quality of life [2]. The clinical presentation is generally divided into three domains: positive symptoms (hallucinations and delusions), negative symptoms (amotivation, anhedonia, and affective flattening), and cognitive symptoms (deficits in memory, attention, and executive function).

While current antipsychotic medications are effective in managing positive symptoms, their efficacy in addressing negative and cognitive symptoms remains limited [3]. These residual symptoms are strongly associated with functional impairment and poor long-term outcomes [3,4]. In addition, many antipsychotics are associated with significant adverse effects, including weight gain, metabolic disturbances (diabetes, dyslipidemia), extrapyramidal symptoms (EPS), and cardiac arrhythmias [3,4]. Some also elevate prolactin levels, leading to hormonal complications [5]. These side effects contribute to poor adherence and high discontinuation rates [1].

To address these limitations, newer agents such as lumateperone have been developed. Lumateperone has been approved by the U.S. Food and Drug Administration (FDA) for the treatment of schizophrenia [6]. It acts through a multimodal mechanism combining serotonin 5-HT2A antagonism, dopamine D2 receptor modulation (postsynaptic antagonism and presynaptic partial agonism), glutamatergic regulation via D1 receptor-dependent pathways, and serotonin reuptake inhibition [6]. This balanced pharmacologic activity restores neurotransmitter function while minimizing off-target effects responsible for metabolic and motor adverse events [2,3]. Studies have shown that lumateperone carries a lower risk of weight gain, EPS, and elevated prolactin levels compared with other antipsychotics [2,3]. This improved tolerability may enhance medication adherence, as patients are less likely to discontinue treatment due to side effects [4]. Additionally, lumateperone is taken once daily, with a half-life of approximately 13 to 21 hours, making it easier for patients to maintain a consistent treatment routine [1].

Despite advances in understanding the biology of schizophrenia, many patients continue to experience limited benefits from available treatments. Approximately one-third of individuals have treatment-resistant schizophrenia, highlighting the need for new therapeutic options with improved efficacy and tolerability. Lumateperone, with its distinct pharmacological profile, has emerged as a promising option for treating schizophrenia [4].

## Review

Methods

A comprehensive literature search was conducted using Google Scholar, PubMed, PubMed Central (PMC), Medline, and the Cochrane Library. The search terms included “lumateperone,” “schizophrenia,” “antipsychotic,” “safety,” “tolerability,” and “adverse effects.” This review follows a narrative approach to synthesize existing clinical and pharmacological evidence on lumateperone’s efficacy and safety. Peer-reviewed articles published in English were included if they specifically evaluated lumateperone in adults with schizophrenia or provided comparative safety or efficacy data versus other antipsychotics. Articles were excluded if a free full-text version of the paper could not be retrieved. Grey literature was not included. After the eligible articles were identified and reviewed, data were extracted to summarize study design, sample size, key efficacy outcomes, and safety or tolerability measures. Because this is a narrative review, no formal quantitative synthesis or risk-of-bias assessment was performed.

Results

Table 1 summarizes the key studies that evaluated lumateperone, including those reporting efficacy outcomes, safety outcomes, or both. It outlines each study’s design, publication year, and sample size, as well as quantitative measures of efficacy and safety or tolerability outcomes (including weight change, incidence of EPS, sedation, prolactin levels, and metabolic effects). The studies range from randomized controlled trials to open-label extensions, providing a comprehensive overview of lumateperone’s risk profile. These results serve as the basis for the clinical considerations explored in the discussion.

**Table 1:** Summary of all studies selected for lumateperone’s efficacy and safety/tolerability outcomes EPS: extrapyramidal symptoms; PANSS: Positive and Negative Syndrome Scale; LS: least squares; SGA: second-generation antipsychotics; NNT: number needed to treat; NNH: number needed to harm; MADRS: Montgomery–Åsberg Depression Rating Scale

Author (s)	Year of Publication	Type of Study	Sample Size	Efficacy Outcomes	Safety / Tolerability Outcomes
Correll et al. [1]	2021	Open-label study	n = 301	Stable PANSS total scores after switch; improvement in metabolic and endocrine markers	Somnolence (9.3%), headache (7.6%), dry mouth (5.0%), rare EPS, ↓ cholesterol, ↓ weight (−1.3 kg mean), ↓ prolactin
Kane et al. [3]	2021	Randomized controlled trial	n = 1073	Pooled analysis confirmed efficacy outcomes consistent with prior phase 3 trials, with symptom improvement comparable to risperidone	Higher completion rate (77.6% vs 65.9% risperidone); lower EPS (3.0% vs 6.3%); smaller mean weight gain (+1.6 kg vs +2.6 kg; p < 0.001); no prolactin increase (–1.3 vs +34.9 ng/mL; p < 0.001); metabolic profile similar to placebo
Correll et al. [6]	2020	Randomized controlled trial	n = 450	Change in PANSS total score was −14.5 with lumateperone 42 mg compared with −10.3 with placebo (p = 0.02), showing significant symptom improvement	Somnolence (17.3%), sedation (12.7%), constipation (6.7%), fatigue (5.3%); extrapyramidal symptoms < 5%; mean weight +0.9 kg vs +0.7 kg placebo; no clinically meaningful metabolic, prolactin, or cardiovascular effects; no serious treatment-related adverse events
Lieberman et al. [7]	2016	Randomized controlled trial	n = 335	Lumateperone 60 mg (n = 76) significantly improved PANSS total score (LS mean change –13.2 ± 1.69 vs –7.4 ± 1.68 for placebo; p = 0.017; effect size = 0.4) and improved social functioning based on a post-hoc analysis of the PANSS-derived Prosocial Factor (–5.0 ± 0.5 vs –2.5 ± 0.5 for placebo; p < 0.001; effect size = 0.59), with benefits across positive, negative, and depressive domains	Placebo-adjusted mean weight gain was approximately 0.3–0.4 kg for lumateperone compared with 2.3 kg for risperidone, indicating substantially less weight gain with lumateperone. No prolactin increase was observed, and sedation and somnolence rates were lower than with risperidone and similar to placebo
Vanover et al. [8]	2019	Clinical pharmacodynamic study	n = 10	D₂ occupancy ≈ 40%; stable psychotic symptoms	Only mild headache or sedation; no extrapyramidal or hormonal effects
Jawad et al. [9]	2022	Systematic review	—	Lumateperone significantly improved positive, negative, and depressive symptoms compared with placebo, with overall symptom reduction across domains	Minimal metabolic or extrapyramidal adverse events; favorable overall tolerability profile similar to placebo
Greenwood et al. [10]	2021	Narrative review	—	Summarized efficacy comparable to that of other second-generation antipsychotics, supported by improvements in positive and negative symptoms across trials	Lumateperone’s multimodal mechanism (5-HT₂A antagonism, glutamatergic modulation, and modest D₂ receptor occupancy) contributes to reduced extrapyramidal symptoms, stable prolactin, and minimal metabolic changes compared with other SGAs
Satodiya et al. [11]	2022	Systematic review	—	Reported consistent symptom reduction across randomized trials and open-label extensions	Mean weight change was minimal (generally under +1 kg in most reviewed trials); metabolic and EPS adverse events were very limited
Zhao et al. [12]	2024	Observational study	—	—	Frequent signals for somnolence, sedation, dizziness, weight gain, and psychiatric manifestations (mania/hypomania); rare but notable reports of suicidal ideation
Citrome et al. [13]	2023	Secondary evidence-based medicine analysis of randomized controlled trials	—	NNT= 8–9 for response (PANSS); benefit–risk favored	NNH high for discontinuation; low overall adverse effects burden
Vanover et al. [14]	2020	Post hoc analysis of a randomized controlled trial	—	Improved social-function domain (PANSS Prosocial factor) compared with placebo, with greater benefits observed in patients presenting with negative or depressive symptoms	Overall well tolerated, with higher somnolence/ sedation than placebo, but no consistent metabolic/prolactin/ QTc signal
Snyder et al. [15]	2021	Narrative review	—	Highlighted lumateperone’s multimodal mechanism (5-HT2A antagonism, glutamatergic modulation, modest D2 effects), low D2 receptor occupancy (~40%) relative to other antipsychotics	Favorable extrapyramidal and metabolic profile compared with standard second-generation antipsychotics
Calabrese et al. [16]	2021	Randomized controlled trial	n = 377	Lumateperone 42 mg significantly improved depressive symptoms vs. placebo on MADRS (mean change −14.7 vs −9.8; p < 0.001) in patients with bipolar I and II disorders	Somnolence and nausea were the only treatment-emergent adverse events occurring at clinically meaningful rates above placebo; overall, well-tolerated with minimal metabolic or EPS effects

As summarized in Table 1, both randomized controlled and open-label studies consistently report that lumateperone provides significant symptom reduction with a low incidence of metabolic and extrapyramidal side effects. Across trials, weight gain was minimal (typically under 1 kg), prolactin levels remained stable, and EPS occurred at rates comparable to placebo. These findings collectively support lumateperone’s favorable benefit-risk balance and provide the foundation for the discussion that follows.

Discussions

Despite 70 years of antipsychotic drug development, most agents have primarily targeted dopamine receptors, with only modest advances in tolerability and control of negative or cognitive symptoms [17]. Lumateperone has emerged as a distinctive antipsychotic due to its multimodal pharmacological action and consistently favorable tolerability profile. Unlike most antipsychotics discovered through dopaminergic screening, lumateperone was advanced through phenotypic drug discovery approaches [18].

The collective evidence from randomized controlled trials and open-label studies demonstrates its potential to address long-standing limitations of antipsychotic therapy, particularly concerning safety, adherence, and broader symptom aspects. Across multiple trials, lumateperone demonstrated significant improvement in schizophrenia symptoms, with effects comparable to risperidone but accompanied by fewer adverse effects [6,7]. Early evaluations also emphasized this favorable balance of efficacy and tolerability, underscoring lumateperone’s potential to improve adherence and overall treatment outcomes [19]. Evidence-based medicine metrics further support this balance. Citrome et al. reported a number needed to treat (NNT) of 8-9 for symptomatic response, with a very high number needed to harm (NNH) for discontinuation due to adverse events, reinforcing lumateperone’s favorable benefit-risk profile relative to other antipsychotics [13]. However, most of these trials were short in duration (typically four to six weeks) and lacked head-to-head comparisons against a range of established second-generation antipsychotics. Consequently, while early findings are encouraging, the generalizability of these results to long-term, real-world treatment remains uncertain.

Lieberman et al. reported that lumateperone at 60 mg improved not only positive symptoms but also secondary measures of negative and depressive symptoms, raising the possibility that its therapeutic benefits extend beyond psychosis reduction [7]. These findings are consistent with the conclusions of Jawad et al., whose systematic review of lumateperone trials found that the drug improved not only positive symptoms but also negative, cognitive, and social functioning domains, while maintaining a favorable tolerability profile [9]. In line with this, an analysis of trial data suggested that lumateperone may improve social functioning, especially in patients with prominent negative symptoms or depressive features, areas often resistant to standard antipsychotics [14]. However, these effects have primarily been observed in short-term studies, and larger, longer-term investigations are necessary to determine if these multidimensional benefits are sustained over time.

Neurological safety represents another area where lumateperone distinguishes itself. Across pooled analyses, EPS were reported at rates similar to placebo [3]. The positron emission tomography study by Vanover et al. provides insight into the mechanism, showing that lumateperone achieves antipsychotic efficacy at relatively low dopamine D2 receptor occupancy, around 40%, compared to the 65-80% typically required for other agents. This lower occupancy helps explain its minimal impact on motor function and prolactin [8]. Greenwood et al. further emphasized that lumateperone’s multimodal mechanism, combining serotonin 5-HT2A antagonism, glutamatergic modulation, and modest dopamine effects, contributes to its favorable tolerability profile and distinguishes it from many other second-generation antipsychotics [10]. These insights into lumateperone’s mechanism of action are consistent with the comprehensive review by Snyder et al., which emphasized its multimodal pharmacology as central to its efficacy-tolerability profile [15]. Kaczmarek et al. highlighted that this broad pharmacological profile has generated interest in lumateperone’s potential applications beyond schizophrenia, although schizophrenia remains its best-established indication [20]. Nevertheless, these mechanistic findings should be interpreted cautiously, as the studies involve small samples and often focus on surrogate endpoints rather than direct measures of functional improvement.

One of the most clinically relevant findings across studies is lumateperone’s minimal impact on weight and metabolic parameters. In both controlled settings and open-label switch studies, patients treated with lumateperone experienced little to no weight gain and, in some cases, demonstrated improvements in cholesterol, glucose, and prolactin compared with baseline or with other antipsychotics [1,3]. This observation is consistent with the systematic review by Satodiya et al., which specifically evaluated body weight outcomes and confirmed that lumateperone is associated with minimal or no clinically significant weight gain [11]. However, as these studies were short in duration, the long-term metabolic impact remains uncertain. This favorable metabolic profile is a significant advantage, given the high burden of cardiovascular disease among people with schizophrenia and the well-established metabolic liabilities of many second-generation antipsychotics.

Sedation was among the few adverse events observed more frequently than placebo, but it was generally mild and manageable. Evidence suggests that administering the drug in the evening substantially reduces daytime sedation, an important consideration for maintaining functioning and adherence [1]. Prolactin levels remained stable in patients treated with lumateperone, avoiding the hormonal and sexual side effects often associated with risperidone and paliperidone [1,3]. Cardiovascular safety is also reassuring, as lumateperone did not meaningfully affect QT intervals or vital signs in clinical trials [6]. While this profile is promising, the absence of long-term cardiovascular outcome data means that sustained safety cannot yet be assumed. Another practical advantage, highlighted by Greenwood et al., is lumateperone’s fixed once-daily dose without the need for titration, which may help patients maintain consistency in their treatment over time [10].

While randomized controlled trials consistently demonstrated lumateperone’s favorable safety profile, emerging real-world data suggest the need for continued vigilance. A recent pharmacovigilance analysis of post-marketing reports found that the most common adverse events associated with lumateperone were sedation, somnolence, dizziness, and weight gain, with psychiatric and nervous system events among the most frequently reported categories. Notably, signals for mania, hypomania, and suicidal ideation were identified, though no signals were detected for suicide attempts or completed suicides [12]. Serious outcomes such as suicidal ideation and completed suicide were also present, although the spontaneous nature of reporting limits causal interpretation [12]. These findings underscore that although lumateperone offers important tolerability advantages in controlled trials, ongoing post-marketing surveillance remains essential to capture rare but clinically significant safety concerns. These real-world findings do not undermine the controlled trial data but highlight the importance of continued surveillance, particularly for rare but serious outcomes.

Limitations

Despite these advantages, important uncertainties remain. The majority of published studies lasted only four to six weeks, limiting conclusions about whether the efficacy and tolerability advantages persist over long-term treatment [21]. Short trial durations make it difficult to evaluate outcomes such as sustained metabolic safety, relapse prevention, and functional recovery, which are crucial in chronic disorders like schizophrenia. Longer real-world and maintenance studies are therefore needed to determine whether lumateperone’s early tolerability advantages translate into lasting clinical benefit. The open-label switch study by Correll et al. showed short-term improvements when patients transitioned to lumateperone, but these benefits reversed after switching back to other antipsychotics, showing the need for sustained treatment data [1]. Furthermore, most trials have compared lumateperone only to placebo or risperidone. Without head-to-head comparisons against other antipsychotics such as aripiprazole, olanzapine, or clozapine, it is difficult to define its place in treatment algorithms. While emerging meta-analytic data have begun to evaluate lumateperone’s efficacy and safety across psychiatric disorders, comprehensive network meta-analyses directly comparing it to a wider range of antipsychotics and studies focusing specifically on cognitive outcomes remain limited. Moreover, the predominance of industry sponsorship introduces potential bias, and independent replication remains limited. 

Another area requiring clarification is dosing. Higher doses have not consistently produced superior outcomes and may heighten sedation instead [7]. This suggests that lumateperone has a relatively narrow therapeutic window, which may be both an advantage in simplifying prescribing and a limitation in terms of flexibility. There is also emerging but inconclusive evidence that lumateperone may improve negative and cognitive symptoms through its modulation of glutamate and serotonin, but larger and longer studies are needed to confirm this effect [7]. Furthermore, lumateperone has shown efficacy in bipolar depression, suggesting its potential utility beyond schizophrenia and highlighting the need for broader and longer-term investigations [16].

## Conclusions

Overall, lumateperone offers a balance of efficacy and tolerability that sets it apart from many existing antipsychotics. Its advantages in metabolic, endocrine, motor, and cardiovascular safety have important implications for adherence and long-term health, particularly in a disorder where treatment burden is already high. Based on current evidence, lumateperone appears most promising for patients who prioritize metabolic and motor safety or who have experienced intolerance to other agents, rather than as a general first-line antipsychotic. While uncertainties remain regarding long-term outcomes, broader comparative data, and its role in treatment-resistant schizophrenia, the existing evidence supports its value as a targeted treatment option within the evolving therapeutic landscape of schizophrenia.

## References

[1] Correll CU, Vanover KE, Davis RE, Chen R, Satlin A, Mates S (2021). Safety and tolerability of lumateperone 42 mg: an open-label antipsychotic switch study in outpatients with stable schizophrenia. Schizophr Res.

[2] Schultz SH, North SW, Shields CG (2007). Schizophrenia: a review. Am Fam Physician.

[3] Kane JM, Durgam S, Satlin A, Vanover KE, Chen R, Davis R, Mates S (2021). Safety and tolerability of lumateperone for the treatment of schizophrenia: a pooled analysis of late-phase placebo- and active-controlled clinical trials. Int Clin Psychopharmacol.

[4] McCutcheon RA, Reis Marques T, Howes OD (2020). Schizophrenia—an overview. JAMA Psychiatry.

[5] Syed AB, Brašić JR (2021). The role of lumateperone in the treatment of schizophrenia. Ther Adv Psychopharmacol.

[6] Correll CU, Davis RE, Weingart M (2020). Efficacy and safety of lumateperone for treatment of schizophrenia: a randomized clinical trial. JAMA Psychiatry.

[7] Lieberman JA, Davis RE, Correll CU (2016). ITI-007 for the treatment of schizophrenia: a 4-week randomized, double-blind, controlled trial. Biol Psychiatry.

[8] Vanover KE, Davis RE, Zhou Y (2019). Dopamine D2 receptor occupancy of lumateperone (ITI- 007): a positron emission tomography study in patients with schizophrenia. Neuropsychopharmacology.

[9] Jawad MY, Alnefeesi Y, Ceban F (2022). Lumateperone for the treatment of adults with schizophrenia: a systematic review. Curr Psychiatry Rep.

[10] Greenwood J, Acharya RB, Marcellus V, Rey JA (2021). Lumateperone: a novel antipsychotic for schizophrenia. Ann Pharmacother.

[11] Satodiya RM, Brown VR, Njuguna SW, Bied AM (2022). A systematic review of clinical trials on lumateperone and its effects on body weight. J Clin Psychopharmacol.

[12] Zhao D, Zhang W, Liu Y, Yan Z (2024). Post-marketing safety concerns with lumateperone: a pharmacovigilance analysis based on the FDA adverse event reporting system (FAERS) database. Front Pharmacol.

[13] Citrome L, Durgam S, Edwards JB, Davis RE (2023). Lumateperone for the treatment of schizophrenia: number needed to treat, number needed to harm, and likelihood to be helped or harmed. J Clin Psychiatry.

[14] Vanover K, Davis R, Durgam S, Huo J, Mates S, Satlin A, Harvey PD (2020). The efficacy of lumateperone 42 mg in the treatment of schizophrenia symptoms associated with social functioning: post hoc analysis of an acute placebo- and active-controlled trial. Schizophr Bull.

[15] Snyder GL, Vanover KE, Davis RE, Li P, Fienberg A, Mates S (2021). A review of the pharmacology and clinical profile of lumateperone for the treatment of schizophrenia. Adv Pharmacol.

[16] Calabrese JR, Durgam S, Satlin A (2021). Efficacy and safety of lumateperone for major depressive episodes associated with bipolar I or bipolar II disorder: a phase 3 randomized placebo-controlled trial. Am J Psychiatry.

[17] Shad MU (2023). Seventy years of antipsychotic development: a critical review. Biomedicines.

[18] Leahy E, Varney M, Brunner D (2020). Use of phenotypic screening in mice in the development of a novel non-D2-receptor-targeting drug for the treatment of schizophrenia. Phenotypic Drug Discovery.

[19] Edinoff A, Wu N, deBoisblanc C (2020). Lumateperone for the treatment of schizophrenia. Psychopharmacol Bull.

[20] Kaczmarek A, Szymajda W, Dettlaff K (2023). Lumateperone in the treatment of psychiatric disorders: a review of the literature. Pharmacotherapy Psychiatry Neurol.

[21] Longo G, Cicolini A, Orsolini L, Volpe U (2023). The novel antipsychotic lumateperone (ITI-007) in the treatment of schizophrenia: a systematic review. Brain Sci.

